# Rethinking the MBA through Hip Hop innovation and Hip Hop innovators: Fat Joe and DJ Khaled pair with two sport × entertainment faculty

**DOI:** 10.3389/fspor.2024.1226233

**Published:** 2024-02-15

**Authors:** C. Keith Harrison, Reggie Saunders, Whitney Griffin, Scott Bukstein, Jeffrey Porter, Brandon Martin

**Affiliations:** ^1^ College of Business, University of Central Florida, Orlando, FL, United States; ^2^ Marketing Department, Nike (United States), Beaverton, OR, United States; ^3^ Psychology Department, Cerritos College, Norwalk, CA, United States; ^4^ Corporate Partnerships, Kansas City Current, Kansas City, KS, United States; ^5^ Athletics Department, University of Missouri–Kansas City, Kansas City, MO, United States

**Keywords:** Hip Hop, MBA program, ethnic entrepreneurship, innovation, creativity, sport management

## Abstract

Theory without relevance for practice in a professional graduate degree has been critiqued by some scholars as a deficit approach when preparing MBA students for the workforce. Scholars and practitioners alike call for more pedagogy in the curriculum with a focus on innovation, creativity, and the involvement of industry practitioners. This paper serves as a case study of a moment in time on Monday, 14 June 2021, when the concept of “pracademics” was realized between two artists and two faculty. Following the transcription of the dialogue between two guest speakers from the Hip Hop world (i.e., Fat Joe and DJ Khaled) and two faculty from the sport management MBA program, the paper analyzes the deeper meaning of their intellectual presence with their expertise in the business of culture (i.e., Hip Hop). Findings reveal how the pairing of Hip Hop artists and faculty in an MBA program can address the innovation gap within global business curricula and models. The broader umbrella of pedagogy and application has implications for other academic disciplines to embrace this concept of pairing academics and industry practitioners.

## Introduction

*From where the pimps, prostitutes, and the drug lords meet*.

*We make a million off of beats, ‘cause our stories is deep.*

Jay-Z, Where I'm from (1).

*Time for Hip Hop to wake up and elevate and dominate the intellectual space*.

–DJ EFN, “Drink Champs” Podcast featuring Michael Eric Dyson (2).

*We getting rich off 16's [vinyl] and can't sing*.

–Dom Kennedy, “Platinum Chanel” lyrics (3).

*Hearing an academic talk about Hip Hop from a completely different perspective is one of the reasons why Hip Hop overshadows all other genres*.

N.O.R.E., “Drink Champs” Podcast featuring Michael Eric Dyson (2).

## Hip Hop as a global culture and business model

Before masterclasses were in vogue, students did not have immediate access to business leaders' wisdom and strategies. In the age of information and virtual gatherings, many business mavens have used digital learning environments to share their innovative approaches with their fields. At the 2021 session of the Founding Fuel’s MBA Virtual Symposium, experts were gathered from the fields of academia and industry to distill a few central themes and questions that are relevant to today's world of business. Each team member was directly involved in management education, either as a visiting faculty member across leading business schools in India or had a leadership role in the management of the schools.

During this symposium panel dialogue of business experts, colleagues agreed that a different design must be integrated with MBA courses in higher education. Consider the following major questions and themes that capture the barriers to innovating many of our current MBA courses:

• If possible, how would you redesign the MBA?
• Engender curiosity in young MBAs.
• Students to self-learn.
• Dismantle the silos of academic disciplines.
• Gen Z has been on lockdown for many years.
• Be eclectic in terms of who gets admission into an MBA program.
• But will these MBA graduates get hired?
• The disconnect is within the economy.
• The academe needs a balance between the required content and when it is required to learn it.
• Why does the MBA constrain the elective course choices of students?
• The academe needs more rigor and relevance.
• The industry needs people with integrative thinking.
• The industry needs job-ready MBAs who can solve problems.
• Who will teach these programs?
• Incentivize practitioners to develop the course.
• Co-teach with academicians and practitioners.
• Give the students time to explore outside the class.

Sport management is the educational platform many students in higher education gravitate toward. Since 1966, such programs in America have increased in quantity and popularity due to student interest, evolving from the physical education model to a business-oriented model (4). Theory without relevance for practice in a professional graduate degree has been critiqued by some scholars as a deficit approach when preparing modern-day MBA students for the workforce and real-world scenarios (5). In order to present graduated students who have demonstrated integrative thinking and problem-solving skills to the workforce, this paper turns to an existing business model that addresses the relevant concerns listed above.

Toppling rock'n'roll as music's top-earning genre, the Hip Hop industry is worth over $15 billion and is the most consumed music genre in the United States (6, 7). As a major business player in the music industry, it encompasses production, distribution, publishing, live concerts, album sales, streaming, and other artform-related activities. In 2017, Goldman Sachs found that for Rhythm & Blues (R&B) and Hip Hop, live music generated $26 billion, publishing generated $6 billion, and recorded songs generated $30 billion (8). For example, in 2018, the largest single-day streaming total for any album on any streaming service to date was due to Drake's fifth album released on Spotify and Apple Music (8). More than just music generation, Hip Hop is a global culture based on five distinct elements, as will be illustrated next.

Exactly 50 years ago, DJ Kool Herc and his sister Cindy Campbell staged block parties in the South Bronx, New York, in 1973. Through a combination of soul, rock, funk, reggae, and Jamaican dancehall toasting genres, dancers and rappers pioneered their art form over looped beats and percussive instrumentations (9). From these informal music scenes in the South Bronx emerged the five elements of Hip Hop: (a) MCeeing, (b) DJing, (c) Graffiti, (d) B-boy/B-girl, and (e) *Knowledge.* An MC stands for “master of ceremonies” and describes a rap performer on stage. A DJ is a “disc jockey” who plays recorded music for an audience, usually on multiple turntables, and introduces the names of the songs on a microphone. Graffiti is an art that is written, painted, or drawn on a wall or other public service, usually without permission. Hip Hop graffiti artists use this form as a means of cultural expression and resistance to hegemony. Just as MCeeing and DJing challenge what counts as music, graffiti challenges mainstream notions of art, public space, and property. Similarly, B-boying/B-girling through breakdancing challenges what counts as dance through shuffles, footwork, poses, and acrobatic moves performed on the floor. The commonality of these elements contributes to the energetic, improvised, and disruptive nature of Hip Hop. Dance floor battles made up of mostly Black and Puerto Rican crew members became renowned for their emphasis on creativity, skill, and musicality.

The fifth element of Hip Hop is knowledge. While it is the element most underutilized in theory and practice, most of the intellectual attention has been rigorously addressed in the social sciences and humanities. This canon has crossed disciplines from sociology, psychology, history, fine arts, dance, kinesiology, African studies, and African American studies (10). From an economic perspective, Hip Hop is worth examining because it occupies a vertical market and has value as an opportunity structure for broader business concepts, e.g., innovation, entrepreneurship, disruption, organizational behavior, and creativity. Groundbreaking partnerships have emerged from the intersection of Hip Hop and sport: Adidas partnered with Run-DMC in the 1980s; Jordan Brand signed endorsement deals with artists Drake, Travis Scott, and DJ Khaled; and rapper Jay-Z founded Roc Nation Sports to manage athletes and execute marketing deals (11). Historically, sports in Black communities (i.e., the post-World War I Negro Leagues) and the Hip Hop industry share a common thread of market disruption: “Both movements exploded out of resistance toward the myth of white superiority in the music and sport industries” (12, p. 207). With few exceptions, most of the content areas covered by the following journals have overlooked, ignored, and outright “dissed” the intellectual value of Hip Hop to build our knowledge base in the academic discipline of business, e.g., with departments typically in management, accounting (13), economics, finance, and sport business.

The case presented in the current study involves industry practitioners and is nested within an MBA course on the intersection of Hip Hop and sport business management. Out of the 213 sport management MBA programs in the United States (14), only one offers a program on Hip Hop entrepreneurship. This aspect of the curriculum introduces an unprecedented pedagogy in the MBA of sport management program design, rendering the case study within it especially valuable for scholars and practitioners who are committed to redesigning the existing structure and curricula.

We want to be intentional before reviewing the literature on rethinking the MBA educational experience; Hip Hop culture and Hip Hop artists are not the only ways of rethinking the MBA (15). In response to the questions listed above, we seek to advance the call for more pedagogy in the curriculum with a focus on *innovation, creativity,* and the *involvement of industry practitioners* through Hip Hop*.* Since the onus to redesign current MBA programs rests on scholars and business experts alike, it would behoove us to analyze a cultural phenomenon that pivoted the business of music, artistry, and ethnic minority representation toward a multibillion-dollar financial gain.

Our positionality in the sport management context is worth noting. While any professor can pair with industry professionals, it was the specific nuances of the Sport × Entertainment faculty in this study that enabled students to openly access the wisdom and strategies of business leaders. Formal programs that foster mentorship and collaboration in higher education can certainly result in innovation. Yet, an institutionalized program may not have precipitated the current pairing, as the second author is neither a full-time professor nor does he have a PhD. What he does have, however, is an adjunct faculty position, full-time professional industry knowledge, and a creative friend. The relationship between the first two authors was the fountainhead of the dialogue presented in the transcript data.

The current paper is focused on applied frameworks for rethinking the MBA through the inclusion of Hip Hop innovators. We note that many of these innovators are Black, Indigenous, and People of Color (BIPOC) individuals and groups who bring unique perspectives to the classroom that enhance student learning and faculty credibility.

## Review of literature

### Gaps in MBA degree programs

Business researchers are immersed in designing and rethinking the MBA degree (16, 17). In their efforts to critically scrutinize the degree and programs, researchers have documented analysis of new and futuristic ideas (18–20); the MBA curriculum at the crossroads (17, 21–25); global/international focus (26, 27); medical and healthcare context (28); human capital (29); women/gender (30); economics (31); industry (32); and cross-disciplinary synergy (33). While our synthesis of the literature is not exhaustive, the topics above are the major themes that scholars have used to contextualize the MBA redesign.

One of the biggest criticisms of the MBA degree in higher education (historically and contemporary at many business schools) is that creative skills are overlooked in the curriculum, are rarely taught, and offer ineffective elective options for MBA students (17). Three major concerns from top MBA programs highlight the necessity of teaching creative skills:

Analytical skills are readily available. People who are creative and innovative are far rarer. MBAs lack creativity. They don't think outside the box. Business schools have to find a way to encourage creativity. Few graduates are capable of formulating “game-changing ideas.” In many sections of the economy, it is innovation and creativity that add the highest value, yet business schools have, at best a modest record of developing these skills. (17, p. 143)

In order to advance research, knowledge, and best practices in sport business, the sports industry should connect with academics to identify solutions to customer retention, service delivery systems, ROI measurement and sponsorship, customer retention, and advertising assessment.

One game-changing idea that emerged in 2021 is the Business of Hip Hop Innovation and Creative Industries Certificate, which is housed in the MBA Sport Business Management program at the [name of university]. This program fills a gap in cross-disciplinary synergy for business schools to encourage creativity by integrating the vertical market of Hip Hop with the economic perspective of business entrepreneurship. While Hip Hop pedagogy is not the only answer to the lack of creative skills, it is a wealth of knowledge for thinking outside the box.

### Creativity in MBA degree programs: ethnic entrepreneurship

Creativity is not an elusive or exclusive character trait. Creativity can be taught to adult learners (34, 35) and business students in particular (36, 37). In their article on enhancing creativity and innovation without encouraging unethical behavior, Baucus and colleagues (38) identified four categories of behavior in business that align perfectly with the rebellious nature of Hip Hop culture: breaking rules and standard operating procedures, challenging authority, avoiding tradition, creating conflict, competition, and stress, and taking risks. Given the lack of creative skills and innovation in 2023, how might Hip Hop culture be framed in a business context to address these gaps in MBA curriculum units? Furthermore, what other non-cognitive skills might Hip Hop culture teach future students?

We see a potential fusion of theory and practice that points to how these gaps in creativity and innovation might be filled. The integration of members of the academic community into the sport industry could result in more strategic, measured, and evaluated systems to deliver sports to consumers. Academics can investigate problems in the business of sports to drive data-informed decisions. Sutton (39) coined the term “pracademics” to merge the two approaches: “That is, academics working with the sports industry and its practitioners to improve the products and services of the industry and increase and retain its consumer base” (para. 13). There is a small body of literature on Hip Hop moguls that contains lessons for business scholars and industry leaders who want to learn from the case studies of Hip Hop catalysts/influencers (40–44). In Watson's chapter from the “Handbook on Wealth and the Super-rich*,*” the commercialization of Hip Hop has provided access to ethnic entrepreneurship:

The mainstream Hip Hop that followed the corporatization of the genre in the mid-1990s would prove to be a huge opportunity for many Hip Hop artists and entrepreneurs, and there are now numerous African American Hip Hop millionaires (45). The so-called Hip Hop “moguls” represent a small number of ethnic entrepreneurs who have risen from the ranks of the many Hip Hop microenterprises to accumulate significant personal wealth, to the tune of hundreds of millions of dollars. They are generally artists who have established successful companies that own the rights to their production, as well as the production of other Hip Hop and R&B artists. Yet they also have business interests that have extended well beyond music. Drawing on the economic power of Hip Hop and their own celebrity status, they have developed business interests in fashion, sport, alcohol and a wide range of other consumer products that appeal to the young consumers of the Hip Hop generation. Some have themselves become iconic brands—cultural icons and representative symbols of globalized Hip Hop culture. (p. 183)

Ethnic entrepreneurship is a means of upward mobility. It is “a set of connections and regular patterns of interaction among people sharing common national background or migration experiences” (46, p. 13). Historically, these entrepreneurs have entered self-employment due to discrimination or entry barriers in the labor market, usually due to their educational background and language deficits (47).

**Figure 1:**
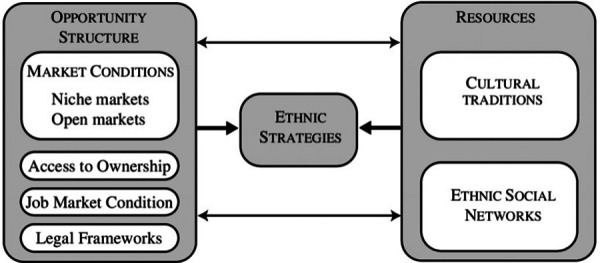
Volery’s (47) ethnic entrepreneurship conceptual model.

The interactive model below illustrates how the success of an ethnic group's entrepreneurial endeavors cannot be attributed to one characteristic; rather, it is the complex interaction between opportunity structures and group resources. The dimension of opportunity structures (on the left) typically materializes from the development of a new ethnic community, e.g., Hip Hop. According to Volery's model, these communities have specific needs that only co-ethnics are capable of fulfilling. Such communities consist of ethnic minority groups or individuals who have immigrated over the past few decades. The greater the cultural gap between the ethnic group and the host country, the bigger the potential niche market. Access to open markets and ownership are often blocked through high-entry financial barriers. The second dimension (on the right) draws from the resources shared by immigrants and ethnic people of the same origin. Cultural traditions can predispose certain groups to assume self-employment. Ethnic networks account for the significance of family and other in-group networks that can compensate for the innumerable disadvantages that immigrants and minorities face in the host country (Figure 1).

The figure above is one snapshot of the many ways scholars, practitioners, and forward thinkers might view Hip Hop through a business/innovation/entrepreneurship prism, especially for racial and ethnic groups who are non-White (48). Ethnic entrepreneurship as a framework enables business scholars as well as practitioners to examine the nuances of ideas that become tangible products and outcomes through the execution of creativity and ingenuity. Before analyzing the presence of two ethnic entrepreneurs in the current paper, we present the relevant research literature that synthesizes the “rethinking the MBA” scholarship line of inquiry.

Based on the synthesis of the MBA degree and ethnic entrepreneurship, the first two authors of the current paper strategized to listen to the business literature's call to take action with theory and practice by inviting two Hip Hop innovators to their classroom (via Zoom lecture platform). The next section describes the method(s) and real-time approach we took with the concept of *pairing faculty* with industry leaders.

## Materials and methods

### Case study and description of participant pairing

**Figure 2:**
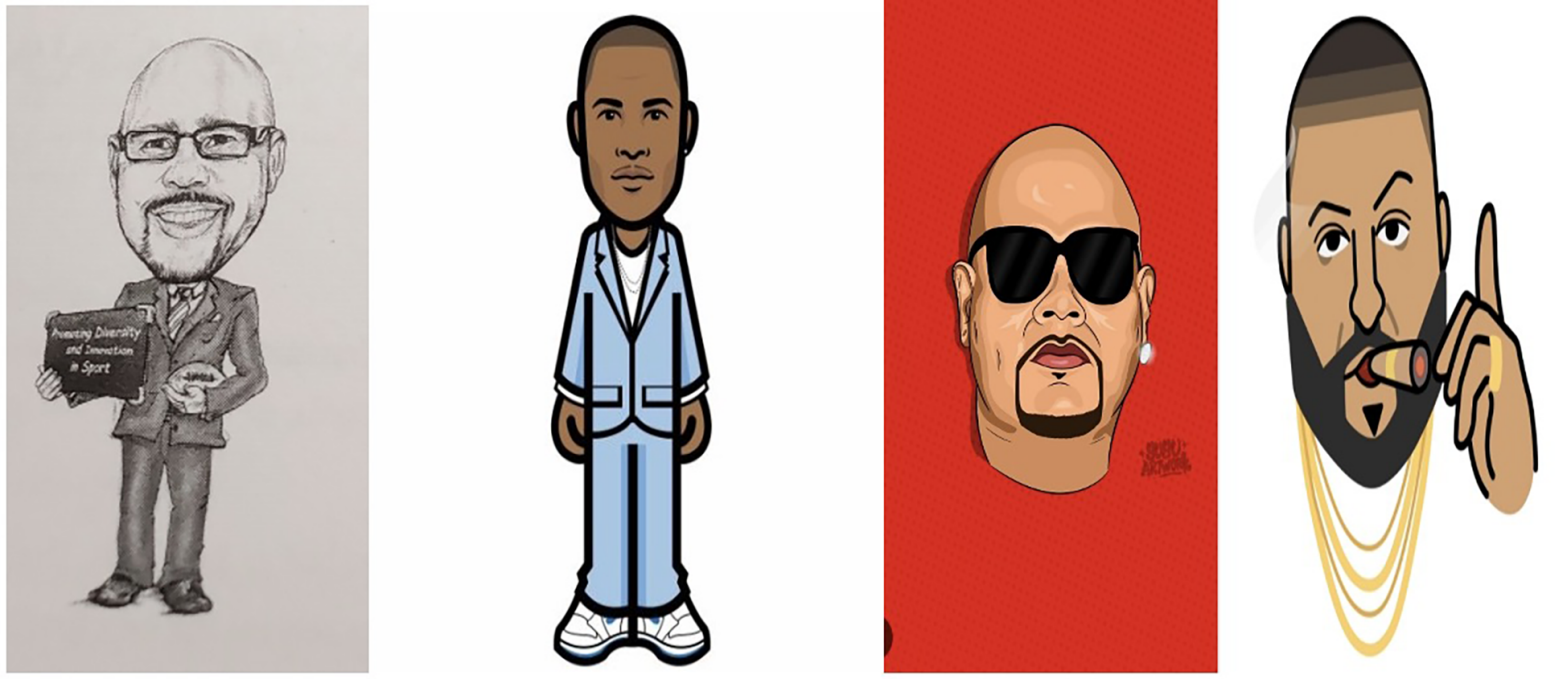
Two professors and two Hip Hop leaders as “Academitioners.”

An important part of any business is human connections within a network of movers and shakers in the industry. An existing business literature has called for rethinking the MBA degree. In particular, scholars and practitioners seek pedagogies that instruct MBA students to think about commerce in futuristic ways to solve timely and timeless challenges. The figure below captures four representative leaders, each of whom brings their own crafts, values, and skills to the intersection of sports, business, and Hip Hop. Combined, these four individuals co-created a novel engagement for *pairing* MBA students with faculty and industry leaders (Figure 2).

From left to right: Keith (professor, founding director of The Business of Hip Hop Innovation and Creative Industries Certificate), Reggie (professor, vice president of Entertainment Marketing for Jordan Brand at Nike), Fat Joe (rapper, songwriter, record producer, record executive, philanthropist), and DJ Khaled (DJ, hype man, rapper, record executive, record producer, philanthropist).

While the two Hip Hop leaders featured in this case study may be most famous for their rap careers, they were included in the current model of ethnic entrepreneurship because of their business acumen. After signing with Atlantic Records, Fat Joe created his own record label called Terror Squad Entertainment, which allows him to sign other artists and distribute their releases. Similarly, DJ Khaled is the CEO of the label he founded under the parent company of Universal Music Group, called We The Best Music Group. He launched his record label to manage, publish, and produce other artists and has released over 10 albums with prominent rappers such as Nicki Minaj, Jay-Z, Future, Chance The Rapper, and Lil' Wayne. Both entrepreneurs have expanded into businesses outside of music. Fat Joe launched *The Fat Joe Show*, wrote a memoir, and donated $100,000 in brand-new clothes to South Bronx students (49). DJ Khaled has partnered with Weight Watchers and Jordan Brand, founded a non-profit organization to provide scholarships for Miami high school students entering the STEAM field, and opened a restaurant franchise named Another Wing with 150 locations across five countries (50). Interestingly, both men have engaged in philanthropic efforts to benefit young students in their native cities, which is arguably the most worthwhile investment that a person can make.

Faculty are tasked with teaching students how to understand the business of sport entertainment. The pairing of academic scholars with industry leaders is not a novel concept. Yet, barriers to innovation in MBA programs persist, and the call for help has intensified. Perhaps participant pairing has not happened enough, or if it has, its effects have not been widely documented. The four leaders in the figure above formed a unique team to simultaneously address several of the valid concerns that were foregrounded in the 2021 session of the Founding Fuel’s MBA Virtual Symposium: (a) engender curiosity in young MBAs, (b) dismantle the silos of academic disciplines, (c) advance the rigor and relevance, and (d) co-teach with academicians and practitioners. The two professors and two Hip Hop leaders paired to produce culturally relevant content in a field of study that is desperate to link theory with practice.

The concept of pairing is a synergistic catalyst that opens many pedagogical possibilities. Not enough professors and faculty understand how to pair properly; they do not move beyond one-time guest speakers, or as scholars, they lack industry knowledge to effectively collaborate. The two Hip Hop innovators for this case study delivered guest lectures in the summer 2021 and spring 2023 courses due to the relationships that the two faculty members had cultivated. Pairing is like a DJ Khaled or Fat Joe song track—many people are involved with some overlapping skills but also unique abilities. Each member in their pair gets their verse, or a turn to display their knowledge, i.e., the fifth element of Hip Hop. And like a Khaled or Fat Joe song, pairing allows for each member to serve as a hype man/hype woman to excitedly encourage, affirm, and energize the intellectual exchange. Tapping into this reality is mutually beneficial. Pairing uplifts many brilliant industry leaders who are foreign to the academic classroom, while faculty benefit by allowing their industry colleagues to *drop knowledge*.

## Transcription of the Hip Hop innovators with MBA and graduate students in sport business management

The following is a transcription of a guest lecture delivered by Fat Joe and Khaled in 2021 for a GEB (General Business) 6156 course titled “The Business of Hip Hop Innovation and Entrepreneurship: Applications to Sport Business Management.” After showing our graduate class the biopic on Fat Joe, he spoke to our students and surprised us with DJ Khaled's presence. The first few exchanges in the introductory remarks are included to contextualize the gratitude for collaboration and appreciation of brotherhood, which are two common tropes in the Hip Hop culture.

### Fat Joe and DJ Khaled industry expert adjunct/guest lecture transcription

[23:42] **Fat Joe**: Oh, you got me. Yo Reggie, you got two for the price of one! I told you I had a surprise. Usually, when you [Professor Reggie] say you have a surprise, it's MJ. I got my Khaled, that's my MJ. You know us, Melissa, chefin' it. Life is beautiful, what you want me to do?

**Keith**: Hey Joe, just appreciate you. I think seven of the eight times Prof Reggie came to teach with me, you called. Khaled heard his voice when he first was pitching to Reggie, so just to you two Hip Hop souls and brothers and leaders, just respect you two and thank y'all for making the time. I just wanted to tell you that first.

**Fat Joe**: Thank you, my brother man. We appreciate this man. All love, always. You know Reggie, one of my best friends on Earth, so there's not nothing I won't do for Reggie. So you know, he calls me 100 times, I'll come 100 times. They know Reggie got Fat Joe in his back pocket.

**Keith**: We spent a semester remixing your “Lean Back” and make it “Lean Up.” We have a whole rap contest. So now the grad students, I'm so glad that they're gonna get access to two of the best in the game.

**Reggie**: Hey Joe, so this is the grad class. It's the intersection of Hip Hop and sport. How would you describe the intersection of Hip Hop and sport? What does each one mean to each other?

**Fat Joe**: Wow, first of all, you can't have one without the other; it just doesn't exist. Even when you go to the Rucker League, the Rucker, the famous street basketball league was formed by rap groups playing against each other. That's how the Rucker started. And then it turned into street basketball where people were playing for real, but it was rap groups against rap groups and vice versa. Basketball is a game of choreography. You know what team is gonna win when you see … it's almost like they're dancing. When you see Trae Young, you see Donovan Mitchell, you see CP3 our guy – I'm so proud of him, he's killing out there, he deserves it – it's a dance, its choreography.

You know when they're in the locker room getting hype for the game, they're playing Hip Hop while they're playing the game; they're playing Hip Hop. Same thing with us, any studio you go into where you see creative minds making music, the basketball is on the screen. Basketball, and all sports to be honest with you, is on the screen. Us, even me, when I work on music, I have like the Jordan documentary. I mean I might say something crazy right now, but I even have the OJ Simpson documentary. All sports legends, and it motivates me to feel like I'm the greatest. I'm fighting for a championship while I'm making music.

**Reggie**: That's like Khaled. Next, you telling me, “Yo this album, I feel like Michael Jordan.” And it was before he even made the album.

**DJ Khaled**: I keep looking at the video every day Reg, still. Me and MJ talking that s**t. There's one thing he said though: “Anything Khaled wants, I’ll green light it.”

**Keith**: Joe, you basically said Hip Hoppers and ballers are trying to ball. So, in other words, performing is balling. Balling, it doesn't matter if you're in the studio or the court, correct?

**Fat Joe**: Whether you play basketball or you make music, creative minds, creatives. Creative. Whoever made the shamguard move, whoever made the cross-over, MJ with the fade away, it's all creativity. Right, and so the creatives think about the people. They have empathy, they think about the fans more than themselves. This is why you see so many of your favorite athlete or artist go through so much. Man DMX, he loved the people so much that he was bugged out because it's a sense – it's a show. CP3 out there, balling putting on a show. Trae Young putting on a show, it's ballet. It's choreography, you can't have one without the other.

Listen, I heard Lil Wayne and 2 Chainz's new album coming up and they got some ballers rapping on there, big ballers. Dame Lillard not the only one, they got big guys. I just saw a song with Lou Williams the other day, vice versa. J. Cole went to the African League to try to ball, it's so interconnected. I watched Dave East play one day, Chris Brown. It's all aligned in one, there's no way –

**Keith** [Question about my former GA (Graduate Assistant Alicia Jeffries) now doing marketing at Detroit Pistons]. They're considering bringing Hip Hop night culture. How can they get it to jump off at any arena? Any advice?

**Fat Joe**: This is a real deep question, I don't know if you want me to answer this truthfully. This is a very – yo, Reggie, stop me if I'm wrong right, if I'm going too deep. Basketball, years ago, they would fine basketball players for dressing up Hip Hop, or getting tattoos, or anything like that. It's different now, they embrace the culture now one million percent. I love how the NBA has supported the Black Lives Matter movement; they've been there with us, one million percent.

*But*, these institutions, these NBA institutions, I guess to keep everybody else other than Black, to keep the White people engaged, they purposely play “Don't go messing with cotton-eyed Joe, cotton-eyed Joe” in these arenas. And I'm wondering who the h**l wants to see the New York Knick dancers dance to “Don't go messing with cotton-eyed Joe”? And I'm like, that's the one area where they need a reality check in every NBA team. They need to embrace Hip Hop dance, Hip Hop culture ‘cause they people. Let's face it, Hip Hop is a natural resource made by American people.

This is a natural resource that's created by Americans that has trickled down trillions of dollars. There's nothing wrong with embracing Hip Hop culture; *fully*, not just one night, fully. When they girls dance at half time and they do three different routines, I don't know anybody in NYC that wants to see—no disrespect—a country dance (begins to demonstrate) but they do it. So, I have a problem with that. Let's just keep it real, White people are going to love the game of basketball no matter what, if you play Hip Hop music or not, and guess what? White people are probably the biggest consumers of Hip Hop music. Whoever is leading that is wrong.

**Keith**: Thanks, Joe, for being honest and having this answer be deeper than the question.

**Fat Joe**: It's all love, brother, it's always love.

**Prof Reggie**: Joe, is Hip Hop a sport in your mind?

**Fat Joe**: Yeah, Hip Hop is competition. It's always been competitive, even amongst friends, even amongst friends. The best rhyme I ever wrote was, “Big L, RIP, his song the enemy.” And because Big L is my little brother and I was just blowing up and we were in the same crew digging in the crates, and I sold half a million records. And he sat me down, in my face: “I'm gonna take your half a million fans. You better bring your A-game cause I'mma do a song with your right now, and I'mma kill you on this song. You better bring your A-game “cause I’m tryna take your fans.” He's the only artist ever told me that in my life. So, I wrote the best rap I ever wrote in my life, on the Big L joint. It's always been competition, no matter what, even if were performing.

If somebody performs before me – I just recently saw that interview with Jay-Z and he said DMX opened up for him somewhere and tore that thing down. He said, “Omg (oh my god) I gotta make some adjustments”. ‘Cause you know, it's competition. It is a sport. The best artist that I ever thought that ever took Hip Hop as a sport the most is LL Cool J. He's my idol, but getting a mic tattooed on his arm, battling every decade; to him, it's really a sport.

On that interview with The Shop, when Lebron James said when he came into the league, there was no Europe step; he had to adapt to the Euro step. Who was the first one to do the Euro Step? I think Gionobli, but now that's common. Joel Embiid, the center (for the Philadelphia 76ers basketball team) is Euro-ing you to death. So, that's what we mean, you are who are you, but you adapt to the way the game is going. If the game is going to 3-point, you learn the 3-point shot and rap music; it just all changes.

A lot of our greats and our legends stay stuck in a certain rhyme pattern and flow or rap to the same type of thing. They don't know how to adjust to what the youth is doing. So, I've been lucky enough to always stay in the studio, always keep the youth around me, always be on point to *adapt* to whatever is moving, and always represent Fat Joe. You could be a fan of Fat Joe for 20 years, you get a new song like “Sunshine.” It's still Fat Joe, but it's modern time and you're like, “Yo this is hot.” It's still Fat Joe, I'm not saying come out tomorrow, drop a record where my voice and everything sounds like The Migos, ‘cause that's the Migos.

**Keith**: Sport takes more from Hip Hop than Hip Hop takes from sport. We'd like to see Hip Hop look more like sport; thoughts on that, Joe?

**Fat Joe**: I think they do it, but I think Hip Hop knows how to hide it more. We're in the studio every single night. You can't go to a strip club where you don't see the basketball game over the bar. You won't go to a studio where you don't see everybody watching the game, so we take the energy from it. We just don't make reference to it all the time, I know I do; tons of rappers do. Khaled watches basketball all day, then he will go and make an anthem like “All I do is win, win, win.” We always do music where we can say you know, they can play this in the arena. That record that he has, the “I did it, I did it, I did it,” that's arena music. The whole influence comes from sport. When Khaled made that record (looks at Khaled and asks if he's right. Asking Khaled) When you made that record you were thinking anthem, arena, – you're thinking sport. We do take away from it, we just know how to disguise it a little bit more.

**Prof Reggie 51**: (Asking about the work ethic he and Khaled had to take.)

**Fat Joe**: It's not like we blew up overnight. That ain't happening. Even this guy here (pointing to Khaled), this guy used to DJ where they gave him $300 and gave pit bulls to watch him, not to secure the club – just to make sure he really DJ'd two hours straight. We had all of our stuff in our car, I've been on tours around the whole country in a van with Big Pun, so everybody is squished. It was Fat Joe and Big Pun, everybody else forget about it and we lived off of McDonald's. But that's the beauty of the journey. When I walked into Khaled's house today and I woke up this morning, you know waking up is half the battle. We wake up like “Ahh man, God let us wake up” and now we go out there to see all the beautiful things we could do and we're so blessed. So, the hard journey will always make us remember how it could be. How hard times could be, and how we have to keep moving forward to make sure we create generational wealth and make sure our families are cared for after we're long gone. The goal is to have great-grandchildren, 80 years from now after we're dead to have a poster of Khaled, to have a painting of Sir. Joseph Carter Jr., Joey Crack, Joseph Crack, and if it wasn't for these guys, we wouldn't be in these type of houses that were in now. *That's the goal*.

**Keith**: (Talking about paying dues and how he wants the students to pay dues.)

**Fat Joe**: I had a Zoom (video call) yesterday and they asked me about the invisible train. Well, I could talk to them about that, or I could talk to them about the real life, something else. I think they have to know if you're in college, if you got a scholarship, God bless you; congratulations. If your parents worked really, really hard to put you in the position that they didn't have, really, really appreciate it. They went all out and they want you to do even better than them and they're proud of you. College ain't ‘Let's just hang out and have a good time.” It's also the last form of where you can have a kid whose family barely scrapped up enough money to put their kid in school, and then you can have somebody whose family owns companies.

I can tell you about Marcus Jordan and his right-hand man who runs his store, Ali; grew up with them in school with Michael Jordan's son. Now they built so much in trust and friendship where Marcus said, alright, be a boss, “I'm bringing my guy with me, Ali.” College is like the last form of bringing two people together that would never be together. Now they're together because they're getting an education, so this is like the one place you can really come up with a friend where the friend says, “No, this is my guy and he's smart,” or “She's my girl, she's smart.” By the way, I never told you my father owns Google and when we graduate, you might have this situation over here. No matter what, you have to be on your best, smartest behavior because this is the last stop for the bridge to gap. Other than that, the rich stay with the rich, and the poor stay with the poor. This is the only time you really intermingle, and you get to meet people who can provide opportunities for you that you don't even know that's there.

To all the students watching, let Fat Joe be your cool uncle, and say this is what your parents are hoping for. They work hard, and they pay your tuition, and you get to mingle with somebody you would've never met if you stayed where you're from. So, you get to bond and you also have to earn their respect. They're not going to be like, “Hey, dad, my friend is the biggest F-up in the world. I met him in college and he drinks 10 liters of beer per second. Let's make him the president of something.” They're not looking for that. You have to be careful. (Turns to Khaled). What you think, Khaled, we on the money with the message?

**Keith**: (Asks about the routine of NBA All-star weekend) – What's the lead up to?

**Fat Joe**: Me and Khaled are like born together, so I don't know how to explain that to you. There's a certain DNA of Terror Squad and We The Best (music group). I'm proud of him, he got his own thing going on. It's a certain DNA we have; goes back to Big Pun, Remy Ma, Khaled, everyone. Reggie was telling us the other day one day, he came to my show, he had his wife and I was rubbing on her belly, she was pregnant. I was being the nicest guy in the world. The minute they said show time, I turned into Fat Joe on stage. We got this DNA where we just did this big show the other day. Some YouTubers were boxing, and Khaled was doing the show and said, “I want you to come.” I come, I know my job. He said, “Alright, you're going to come out here.” I rip it down, he ripped it down, and then we went to eat dinner.

We've been doing this for so long that the energy is just built inside of us. We know we get paid to make the crowd go crazy and into a frenzy. We get paid to have people go home and be like, “Did you see Khaled? Did you see Joe?” And so we give them everything, and we leave it all on the stage. Me and DJ Khaled never take shortcuts when it comes to that, when we're on stage, we give them one million percent. We never went in there one time and cruised. You know when you see a boxer fighting a fight and you think, man Shane Mosley, you could've gave them a little more. He was just cruising it, making it to the 12th, we never do that. We go in there and we try to kill the whole place, no matter what.

**Keith**: What does it mean to know now that Hip Hop and business innovation is in the curriculum?

**Fat Joe**: It's a deeper conversation, brother. It's a deeper conversation and it's something that we have to do a real forum on. The music game has been taking advantage of artists for many, many years. I don't know how it's been so – me and Khaled were very, very blessed. I don't want this to sound like reparations, not reparations, but what about the artists that are struggling? You know, some record labels, you can find a mail room clerk that has a 401k; but the artists, the creatives, the talent that make the music don't even have health insurance. I know it's like, you work for yourself, you're self-paid, but we have to try. We can't walk back and go back to the labels and tell them, “Yo, pay us, you robbed us.” Moving forward, there has to be a way we can help the artists, moving forward to not get taken advantage of. That's a whole different forum, we have to sit down with the smartest minds, ‘cause I ain't the one. We have to sit down with the brother who got every Ph.D. in the world who loves Hip Hop and says, ‘You know what? I don't want to see the Five Heartbeats story, I don't want to see the Hector Lopez’, you know what I mean? That fell through the cracks. Look at you, you do a class on Hip Hop. So, I'm saying how many lawyers are out there, credible lawyers, that would love to donate some time to helping their favorite rapper? I don't want to say names, but whoever. To get some healthcare, I mean that's the conversation that we need to have. And I don't know how to have it, but it's something I really want to dedicate my life to, soon.

**Keith**: (Talks briefly about Professor B's twin brother picking the Sun's team, first class at a graduate level teaching Hip Hop, and how the graduate students should be learning something from Doc, Prof Reggie, and Fat Joe flow).

**Fat Joe**: You want to hear some controversial stuff without cursing? America has been going abroad taking natural resources from others countries since the existence, may it be oil, may it be gold, may it be whatever. Hip Hop was created here, it is American. It is a natural resource that has provided millions and millions of jobs. We have to protect the art form and the artists, the creatives, because you know what? Just because you're a genius at one thing, doesn't mean you're a genius at everything else. So, Fat Joe could be the illest, you could say Fat Joe made “Lean Back,” “All the way Up.” Fat Joe ain't the best accountant, so we have to protect this guy, get this guy an accountant that cares. That's what I'm talking about. It's a deep discussion, that I'm willing to have down the road and I really want to dedicate my life to like, trying to help others. I was blessed to blow up, lose it all, blow up, lose it all, blow up, and I plan to stay up. It's only when this happens, because let's be honest, Michael Jordan might not really know how his best friends really are because he's always been the biggest guy in the world. You don't know how your true brothers and sisters are until you fall. When you fall is when you see everybody running for the border: “I have to get with the next program, oh this guy is done, he ain't got no more money, he ain't hot no more.” Then Joe Crack comes back, boom, so now I get to see who is the real deal and who's not. That's why I try to tell you, there's no sure thing as failure, there's only lessons. God is showing you, “Yo Joe, you don't need these people around you.” Who is with you? They were with you when you said you ain't have no more money, that's who you need with you. Other than that, you keep it moving. I'm proud to say that Reggie has been there with me the whole way! I will tell you, I've been in my darkest moments and Reggie will say. “I believe in you brother. Keep going man, you got this, you got this,” And look, we're back.

**Reggie**: When Joe went away I would write him back, and we had to figure out what he needed to be good.

**Fat Joe**: God is good man, and we're sticking to the program.

**Reggie**: (Asking Joe and Doc for any last words before they go eat).

**Fat Joe**: I ate some fish my wife made me and I came to hang out with Khaled because you know, Khaled will mess up the whole day in a good way. He will call you in the morning and be like, “Yo, what are you doing? And you think you're going to work out? You think you have a whole plan?” And he says, “Alright, I'm over there, I'm on my way,” and we just vibe.

## Q&A from the MBA/MSBM cohort at UCF (summer 2021)

### Question 1

#### What is the biggest lesson you learned while being at the bottom in the Hip Hop industry?

**Fat Joe**: I'm a unique DNA. So, when I grew up, I grew up with nothing. And just like 50 Cent said, I was going to get rich or die trying. So, I am of a species that will never give up, no matter what. Even if you're looking at me and you're saying this guy is crazy, he'll never do it again, no way—I always bet on myself. So, as long as I was alive and I was healthy, I knew there would be some sort of comeback. To be honest with you, most people that are successful—not just in rap music, in any kind of career; you can be a baker, you can be a lawyer, a mechanic—you always know you have what it takes to be successful and go to another level. You have to figure out what is the system for you to help you get to the next level.

### Question 2

#### How do you find the balance when you're at the top—to not let that get too high in your head?

**Fat Joe**: Well, me, I don't use drugs. So, the only way I get high is through success. It would be similar to somebody smoking crack or something. It touches that part of the brain for me where I'm like, “Oh my gosh, everyone is playing my music, it's hot.” That gets me high. Now, I give every artist a year, two-years grace period. Say you, yourself (the person who asked the question) become famous, and all of sudden, all the girls you ever looked at and thought were beautiful in your life all of a sudden start dm'ing (direct messaging) you and running up to you and telling you you're handsome – I mean it's a lot to deal with. So, for me being in the game so long, I give a new artist a year, two years to really know who their true self is because after that, the high is over. After that, you're either humble or a terrible person; and we'll figure it out in two years. Either or, and so you have to check yourself. There is no person who hasn't once or twice in their life gotten so successful that they start thinking their sh** doesn't stink. Then, you start thinking that you made this happen, so you aren't taking advice from anyone else ‘cause you know everything – which is not true.

### Question 3

#### Do you feel a deeper connection to your community, and what are some of your favorite ways to give back?

**Fat Joe**: You know my community has always been crime time, in good or bad time. I come from the projects, I come from welfare—a struggling family. I always think of community: that's why I open businesses in my community, constantly giving food, computers to school, sneakers to school. We sent four airplanes, a million pounds of food and water, women's hygiene to Puerto Rico in the hurricane. I could sit here and tell you like, guys like Fat Joe are rare because if I won the lotto, the whole hood would win the lotto. That's why I'm scared that I'll never win the lotto because I'm not the type of guy that they let win the lotto. I'm all about my people, I'm all about sharing with my people, I'm all about sharing with my community. We're going Thursday, me and Khaled's wife, were going protesting. Some kids contacted her, they said they're trying to build a new school on their playground. And in the Bronx, they have the most Asthma, the most diabetes, and juveniles, this and that. We're literally going to protest Thursday with the kids. We do this all the time when the cameras ain't on. We always engage without our community, you know I'm nothing without my community, to be honest with you. (40:41)

**Student at UCF sent to the first author,** 2022**:** My name is DP and I have been a student in your classes throughout my journey at UCF. I wanted to thank you for introducing the Business of Hip Hop program at UCF. Taking these classes really has changed my life and I have finally found that I truly have a passion for Hip Hop, and this is the route that I would like to take when I graduate. I want to continue to contribute to Hip Hop in the business field and shed light on the originality and creativity of Hip Hop. I am reaching out in regards to asking if you have any knowledge on ways that I can get more involved in Hip Hop as well as expand my knowledge and experience such as internships, etc.? I would really appreciate your insight and I look forward to hearing from you soon.

## Discussion

The type of industry success that Fat Joe and DJ Khaled have enjoyed as Hip Hop artists made them especially relevant for the MBA in the current study. Due to the program's focus on innovation and creativity, Fat Joe and DJ Khaled have consistently released records featuring other artists. Their real-world business partnerships have allowed them to rethink Hip Hop and advance the collaborative nature of the culture. In his memoir, “The Book of Jose,” Fat Joe praised his colleague for his partnerships:

Khaled's projects are part compilations, part soundtracks, and all all-stars. My brother has been able to parlay his relationships into recruiting everyone from Jay-Z and Beyonce' to myself, Rock Ross, Drake, Busta Rhymes, Chris Brown, Lil Wayne, Nicki Minaj, Justin Bieber, T-Pain, Nas, and Rihanna to record for him. He then weaves in all into a cohesive body of work (51).

The relationships that DJ Khaled has parlayed have allowed him to combine artists and genres in novel ways. Khaled's net worth of $510 million supports his accomplishments as an entrepreneur (52). His successful execution of creativity and ingenuity make him an ideal candidate to pair MBA students with game-changing practitioners.

The case of Fat Joe and DJ Khaled's pairing contributes to the research on rethinking the MBA. First, it addresses the innovation gap within numerous MBA curriculum programs. The presence (and knowledge) of Fat Joe and DJ Khaled guest lecturing with approximately 20 MBA/MSBM students demonstrates the strengths of pairing faculty with industry leaders/executives/influencers/catalysts. Theory becomes a practice in real-time, a conversion that research literature has encouraged scholars to consider (53, 54).

The second contribution is that this case study pairs industry leaders from the business world (e.g., sports/entertainment) with faculty in higher education. The major themes from their guest lecture on their industry knowledge created a platform for major takeaways that, at a minimum, engaged MBA students in learning in different ways from traditional instruction. From our case example and moment of pairing (Summer Session A 2021), the faculty and graduate students asked questions that were engaging, non-linear, and based on experiential knowledge (55). Furthermore, this method of engagement provided accessible means of knowledge expression by designing options in the curriculum for multiple media for communication. Rather than relying on textbooks as the sole source of information, students interacted with Fat Joe and DH Khaled as an added option for constructing knowledge of ethnic entrepreneurship. Providing multiple means of action and expression is an equitable teaching practice within the Universal Design for Learning, a research-driven framework to guide the design of accessible learning environments (56).

Third, this case study highlights the intersectionality of Hip Hop and sports for MBA and other graduate students in sport management to think about *creativity and innovation* (57, 58). This intersection of artists and athletes mirrors one another culturally, e.g., fashion, art, and style. Industry practitioners increasingly see the relevance of the intersections of sport (and also sport management) and entertainment. The Sports Business Journal created a 2-day event called “4SE” (https://4-se.com/), which is one relevant example of the intersection between sport, lifestyle, culture, and entertainment. This multi-industry innovation marketed their 2023 event with “Meet our 2023 innovators” featuring Fat Joe to engage fans and serve their communities. One of the criticisms of MBA programs listed at the beginning of this paper is that the industry needs people with integrative thinking. Sports Business Journal events, such as the 4SE, foreground conversations that are at the root of the industry shift and would give MBA students time to explore cross-cultural innovation outside of the class.

Other contributions (not all will be predictable in the future) by the current case example of the “pairing” of two faculty members with Fat Joe and DJ Khaled are key when considering the 4SE concept(s). In fact, Fat Joe is one of the invited speakers at the 2023 4SE event, and Hip Hop innovators will continue to be invited to spaces such as higher education and other platforms often overlooked in the past years. Part of innovation theories is connecting the dots in ways that previous scholars and practitioners overlook (17). One successful example of this formula is the collaboration between Martha Stewart and Snoop Dogg on a popular TV show. While initially perceived as two divergent celebrities and personalities, this show merged generations, gender, race, cooking, and other aspects of culture and lifestyle.

Finally, some researchers have theorized that Hip Hop culture, rap music, and its artists deliver genius and intellect to the higher education game of learning (59). The current paper serves as one case that demonstrates a valid effort to capture this genius with the four “academitioners.” The collaborative pairing was designed to challenge students to think *critically and innovatively with purpose*.

Limited research runs counter to the current paper's positive approach to pairing the industry with faculty from any genre. However, there are documented challenges with pairing (5). Some public observers are reticent about the presence of Hip Hop in higher education (60). There are also several limitations within the current case study. First, we only investigated the pairing of four people. Second, those participants were only located within one discipline. Future research should expand beyond the sport management realm and analyze pairing transcripts from medical innovators, educational psychology innovators, and other fields that need to engender curiosity in problem-solving.

### Outlook

The synergy of Hip Hop innovators and educators is full of potential. More pairing between industry leaders and faculty is encouraged when redesigning MBA programs. It may happen because of the existing relationships and networks. Yet, it is important to note that preexisting relationships are not a requirement for the synergy described here. For example, when a course on Nicki Minaj was announced at UC Berkeley for the Spring 2023 semester, the artist responded on Twitter that she would show up for it (61). For a course that centers on Nicki Minaj, Hip Hop, and feminism, a professor could only design the curriculum from an outsider's perspective. If they functioned as a pair, the students would gain access to the relevance of feminism, economic structures, and the broader sociohistorical context of Hip Hop from an industry insider. Beyond the success of her musical artwork, Nicki Minaj is capable of speaking to her multiple entrepreneurial endeavors that include starting her own management company and record label, releasing a perfume as a companion fragrance to her album, and (perhaps most relevant to the current study) becoming the global ambassador for a sports betting lifestyle brand called MaximBet. Faculty and curriculum designers should think about other cross-cultural collaborations across ethnic, racial, and gender identity pairings.

The table below provides a list of the growing courses, research institutes, and fellowships that have sprung from notable Hip Hop artists. This list is relevant to the current case study because it illustrates the potential for participant pairings.

## Conclusions: 27 Summers “Johnny Nunez got all the pictures”

We want our last section to demonstrate how to rethink the MBA in real-time. The current research began with a list of challenges collected from our colleagues and “intellectual industry homies.” The first and second authors of the current paper have worked diligently for this to occur, and this is how the dots connect as we flesh this out through Nas's 27 Summers video from (62).

Keith and Reggie of the current paper have been team co-teaching in tandem with undergraduates and MBA students since 2012. Keith is a Nasir Jones Hip Hop Fellowship alumnus at Harvard University's Hutchins Center for African and African American Research. Khaled has delivered guest lectures in the courses taught by Keith and Reggie on the business of Hip Hop. Keith has included Steve Stoute's book (63) titled “The Tanning of America: How Hip-Hop Created a Culture that Rewrote the Rules of the New Economy” in his course on innovation and entrepreneurship in the field of sport/entertainment. Johnny Nunez, who was mentioned in the “27 Summers” video before the golf interlude, has also guest lectured for Keith and Reggie in their Hip Hop business course. Professor Todd Boyd from USC has always said, “Do not just talk about it; be about it.” That is the goal and message of this paper, in part based on theory and practice.

Nasir Jones, better known as “Nas” took nearly three decades to win a Grammy—but he *did it*. In the middle of his “27 Summers” music video, Nas and his colleagues (Steve Stoute, DJ Khaled, and another peer are all chilling on the golf course) capture what many African American communities deem as “Black Excellence”:

**Steve Stoute**: My man knows what he's doing. 27 summers in a row … of being relevant.

**DJ Khaled**: It don't matter. The summer is ours every year.

**Steve Stoute**: True story.

**DJ Khaled**: Nah, real talk.

**Steve Stoute**: Doing your thing at a high level, it's called timeless.

**DJ Khaled:** That's all we do is win. “King's Disease” is just like the beginning of the decade, this the new chapter. What we did before has been done and Nas is living proof of it.

**Steve Stoute**: Cheers, guy. Let's go hit a few (golf balls).

This pause/interlude during Nas's 27 Summers video (62) captures in part the success of Hip Hop culture as a mirror 50 years later since its inception at 1520 Sedgwick Avenue in the Bronx. The resilience of Hip Hop a half-century later has found another niche: American higher education. Fellowships named after Hip Hop artists such as Nas, graduate and undergraduate courses on Hip Hop, and Hip Hop artists' presence continue to increase at universities in North America (see Table 1 above). The first course ever taught on Hip Hop was documented at Howard University in 1991.

**Table 1:** Select courses in Hip Hop that use artists as case studies.

Course title	Hip Hop artist	University
Course: “The Galaxy of Hip-Hop Feminisms”	Nicki Minaj	University of California, Berkeley
Course: “The Marathon Continues: Building Brand Through Culture”	Nipsey Hussle	Loyola Marymount University
Course: “Deconstructing Drake and The Weeknd”	two artists’ journeys to the top at Ryerson University	Toronto Metropolitan University
Course: “The Power of Us, It's Bigger than Hip Hop”	Kendrick Lamar	Concordia University
Course: “Cardi B and Respectability Politics: Intersectionality of Race, Gender, and Sexuality”	Cardi B	University of California, Los Angeles
Course: “Black Women, Beyoncé, and Popular Culture”	Beyoncé	University of Texas at San Antonio
Course: “Kanye vs. Ye: Genius by Design”	Kanye West	Concordia University
Course: “The Prophetic Witness of Tupac Shakur”	Tupac Shakur	Boston University
HipHop Archive Research Institute, and Hutchins Center for African and African American Research Fellowship Project: Carry on Tradition: From Nas to Nipsey the Business Education, Innovation and Entrepreneurial Hustle	Nas Nipsey Hussle	Harvard University

These are only the 9 select courses listed, and only those courses are dedicated to a single artist per campus versus courses that cover the broader Hip Hop culture.

In the final analysis, Hip Hop innovation and Hip Hop innovators are only one way to rethink the MBA and deliver what our students need to be competitive in an ever-changing workforce. Hip Hop is an important way for interdisciplinary fields to think differently, not just in the realm of business but also in the domain of education. Peace.

## Data Availability

The original contributions presented in the study are included in the article/Supplementary Material; further inquiries can be directed to the corresponding author.
